# Synthetic correlated diffusion imaging hyperintensity delineates clinically significant prostate cancer

**DOI:** 10.1038/s41598-022-06872-7

**Published:** 2022-03-01

**Authors:** Alexander Wong, Hayden Gunraj, Vignesh Sivan, Masoom A. Haider

**Affiliations:** 1 Department of Systems Design Engineering, University of Waterloo, Waterloo, Canada; 2 Joint Department of Medical Imaging, Mount Sinai Hospital, Princess Margaret Hospital, University of Toronto, Toronto, Canada; 3 Ontario Institute for Cancer Research, Toronto, Canada; 4 Lunenfeld-Tanenbaum Research Institute, Toronto, Canada

**Keywords:** Prostate cancer, Magnetic resonance imaging, Cancer imaging

## Abstract

Prostate cancer (PCa) is the second most common cancer in men worldwide and the most frequently diagnosed cancer among men in more developed countries. The prognosis of PCa is excellent if detected at an early stage, making early screening crucial for detection and treatment. In recent years, a new form of diffusion magnetic resonance imaging called correlated diffusion imaging (CDI) was introduced, and preliminary results show promise as a screening tool for PCa. In the largest study of its kind, we investigate the relationship between PCa presence and a new variant of CDI we term synthetic correlated diffusion imaging (CDI$^s$), as well as its performance for PCa delineation compared to current standard MRI techniques [T2-weighted (T2w) imaging, diffusion-weighted imaging (DWI), and dynamic contrast-enhanced (DCE) imaging] across a cohort of 200 patient cases. Statistical analyses reveal that hyperintensity in CDI$^s$ is a strong indicator of PCa presence and achieves strong delineation of clinically significant cancerous tissue compared to T2w, DWI, and DCE. These results suggest that CDI$^s$ hyperintensity may be a powerful biomarker for the presence of PCa, and may have a clinical impact as a diagnostic aid for improving PCa screening.

## Introduction

Prostate cancer (PCa) is the second most common form of cancer in men worldwide and the most frequently diagnosed cancer among men in more developed countries, with roughly 1.4 million new cases in 2020^1^. While the overall 5-year survival rate for prostate cancer is very high, prognosis is poor for patients with distant metastases outside of the prostate^2,3^. As such, early diagnosis of PCa is critical for improving the treatment of patients with PCa. Clinical screening for PCa has traditionally involved the use of prostate-specific antigen (PSA) screening, with high PSA levels used as an indicator of PCa^4^. Unfortunately, studies have shown that PSA screening has led to a significant over-diagnosis of men suspected of PCa, resulting in over-treatment that carries significant risks^5,6^.

While diagnostic imaging has been increasingly prevalent for PCa screening and diagnosis, one can argue that there is currently no universally accepted method for screening and diagnosing prostate cancer via imaging. Transrectal ultrasound (TRUS) is routinely used to guide prostate biopsy; however, its use for PCa screening and diagnosis is limited due to the fact that PCa tumors are often isoechoic and thus cannot be delineated from surrounding tissue via TRUS. As a result, PCa screening and diagnosis using TRUS has low sensitivity and specificity^7^. PCa screening and diagnosis using positron emission tomography (PET) has also been explored, with several tracers showing promise for delineating cancerous and non-cancerous tissue in the prostate gland^8–11^. Unfortunately, the high cost of PET scanning makes it impractical as diagnostic tool early in the screening pathway.

Magnetic resonance imaging (MRI) has grown significantly in prevalence for the purpose of PCa screening, with wide acceptance of the standardized Prostate Imaging Reporting and Data System (PI-RADS)^12^. T2-weighted MRI (T2w) has been well-studied for PCa screening and diagnosis^13–16^, where potentially cancerous regions are characterized by signal hypointensity, and is considered the primary determining modality for the transition zone (TZ) in PI-RADS^12,16^. However, T2w signal hypointensity in the peripheral zone (PZ) of the prostate gland can also be associated with a number of non-cancerous abnormal conditions such as inflammation, fibrosis, and hemorrhage^17,18^, leading to false positives if T2w was the sole method used. To improve diagnostic accuracy when using MRI for PCa screening and diagnosis, two complementary MRI techniques have been leveraged for improved PCa screening alongside T2w: (1) diffusion-weighted imaging (DWI) with apparent diffusion coefficient (ADC) calculated from DWI, and (2) dynamic contrast-enhanced (DCE) imaging^17^. These techniques, when used together with T2w, form a multi-parametric MRI (mpMRI) strategy to overcome the shortcomings of each modality. However, the need to interpret several modalities can increase interpretation challenges, resulting in increased inter- and intra-observer variability.

Recently, a new MRI technique called correlated diffusion imaging (CDI)^19^ was proposed for improving PCa diagnosis. Preliminary studies demonstrated the potential of CDI for delineating between cancerous and non-cancerous tissue^19,20^. However, the scope of these studies are limited in terms of patient cohort size and diversity (e.g., a patient cohort of 20 patient cases^19^). Furthermore, a number of limitations exist in CDI as first introduced with respect to signal-to-noise ratio (SNR) and acquisition time, as well as SI variability amongst inter-patient and intra-patient acquisitions.

The contribution of this study are twofolds. First, this study represents the largest study of its kind for exploring the relationship between PCa presence and CDI signal hyperintensity across a cohort of 200 patient cases. Second, we introduce an extended variant of CDI we term synthetic correlated diffusion imaging (CDI$^s$), which leverages a hybrid of native and synthetic diffusion signal acquisitions and signal calibration for greater consistency in dynamic range across machines and protocols. We compare the performance of CDI$^s$ for PCa delineation to current standard MRI techniques (T2w imaging, DWI, and DCE imaging). This study aims to provide insights on the potential clinical impact of CDI$^s$ as a diagnostic aid for improving PCa screening.

## Results

In this study, we investigated the efficacy of CDI$^s$ from two different perspectives. First, we studied the relationship between CDI$^s$ SI and the presence of PCa, both clinically significant PCa (csPCa) tissue and clinically insignificant PCa (insPCa) tissue. Consistent with the contemporary concept of csPCa versus insPCa^21^, csPCa tissue is defined as tissue with a Gleason score greater than or equal to 7 (Gleason Grade Groups 2-5 according to the International Society of Urological Pathology) while insPCa tissue is defined as tissue with a Gleason score less than 7 (Gleason Grade Group 1). Second, we studied the performance of CDI$^s$ in delineating csPCa tissue and insPCa tissue from healthy tissue.

### Relationship between CDI$^s$ SI and the presence of PCa

**Figure 1:**
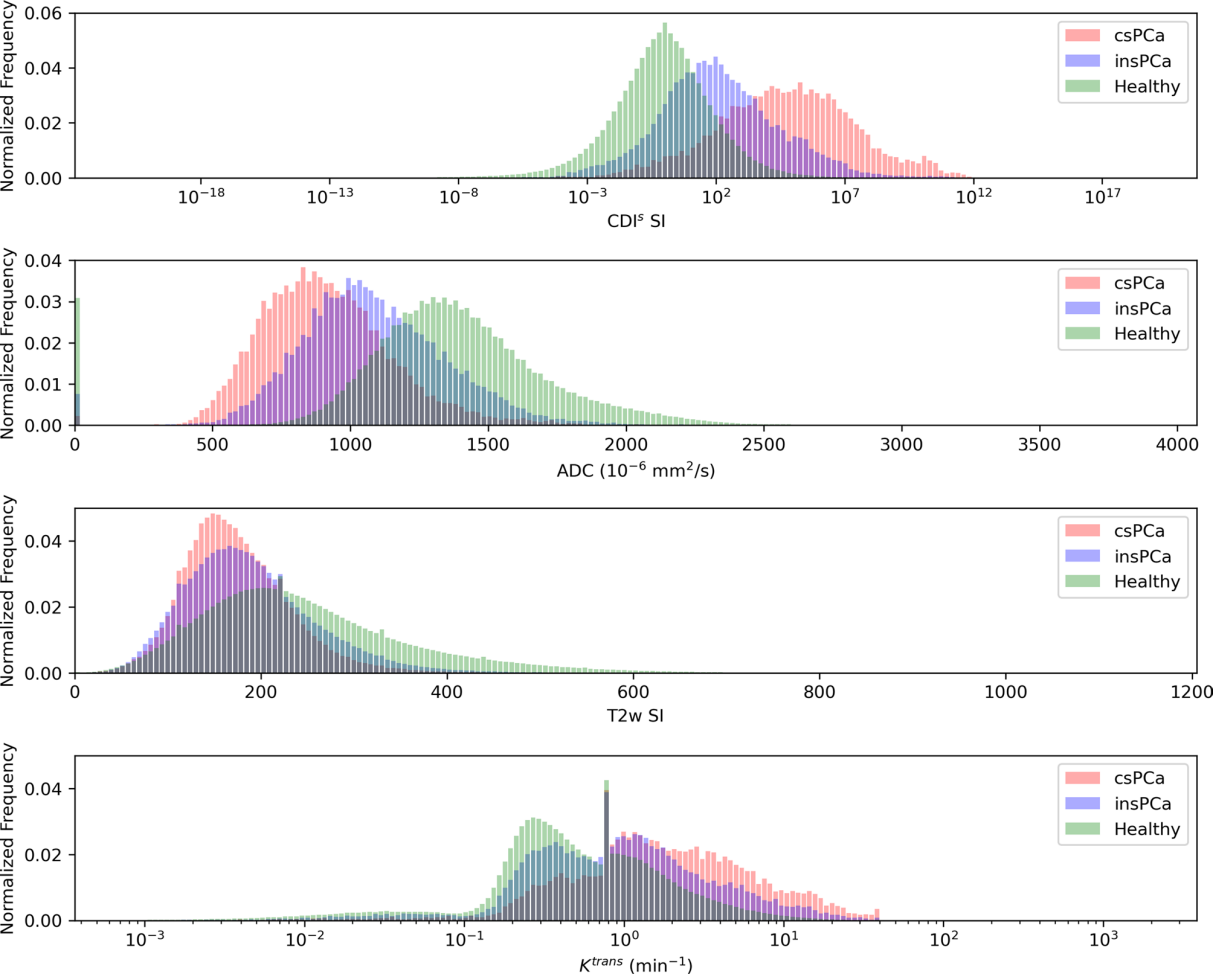
Histogram analysis of CDI$^s$ SI, T2w SI, DWI-derived ADC values, and DCE-derived $K^{trans}$ values for healthy tissue, insPCa tissue, and csPCa tissue.

Figure 1 shows the histogram analysis conducted to study the distribution of CDI$^s$ SI, T2w SI, DWI-derived ADC values, and DCE-derived $K^{trans}$ (volume transfer constant) values for healthy tissue, csPCa tissue, and insPCa tissue. A number of observations can be made from this histogram analysis. First, CDI$^s$ SI hyperintensity is clearly exhibited in the presence of csPCa, with the clinical significance of PCa (from healthy tissue to csPCa tissue) progressively increasing with the CDI$^s$ SI. This observation means that not only can CDI$^s$ SI hyperintensity be a good indicator for the presence of PCa, but can also be a good risk assessment and treatment planning tool for quantitatively assessing the degree of disease severity. Second, it can be observed that there is noticeably lower overlap between the CDI$^s$ SI distributions of csPCa tissue and insPCa tissue when compared to that of T2w SI, DWI-derived ADC values, and DCE-derived $K^{trans}$ values. More specifically, the T2w SI distributions of healthy tissue, csPCa tissue, and insPCa tissue all have considerable overlap, while the value distributions of insPCa tissue and csPCa tissue have greater overlap for both $K^{trans}$ and ADC values. This observation means that CDI$^s$ can potentially be a good clinical decision support tool for clinicians when compared to the current standard MRI techniques in determining the course of action for a patient, be it watchful waiting, active surveillance, or immediate treatment.

### Delineation between PCa tissue and healthy tissue based on quantitative analysis

**Figure 2:**
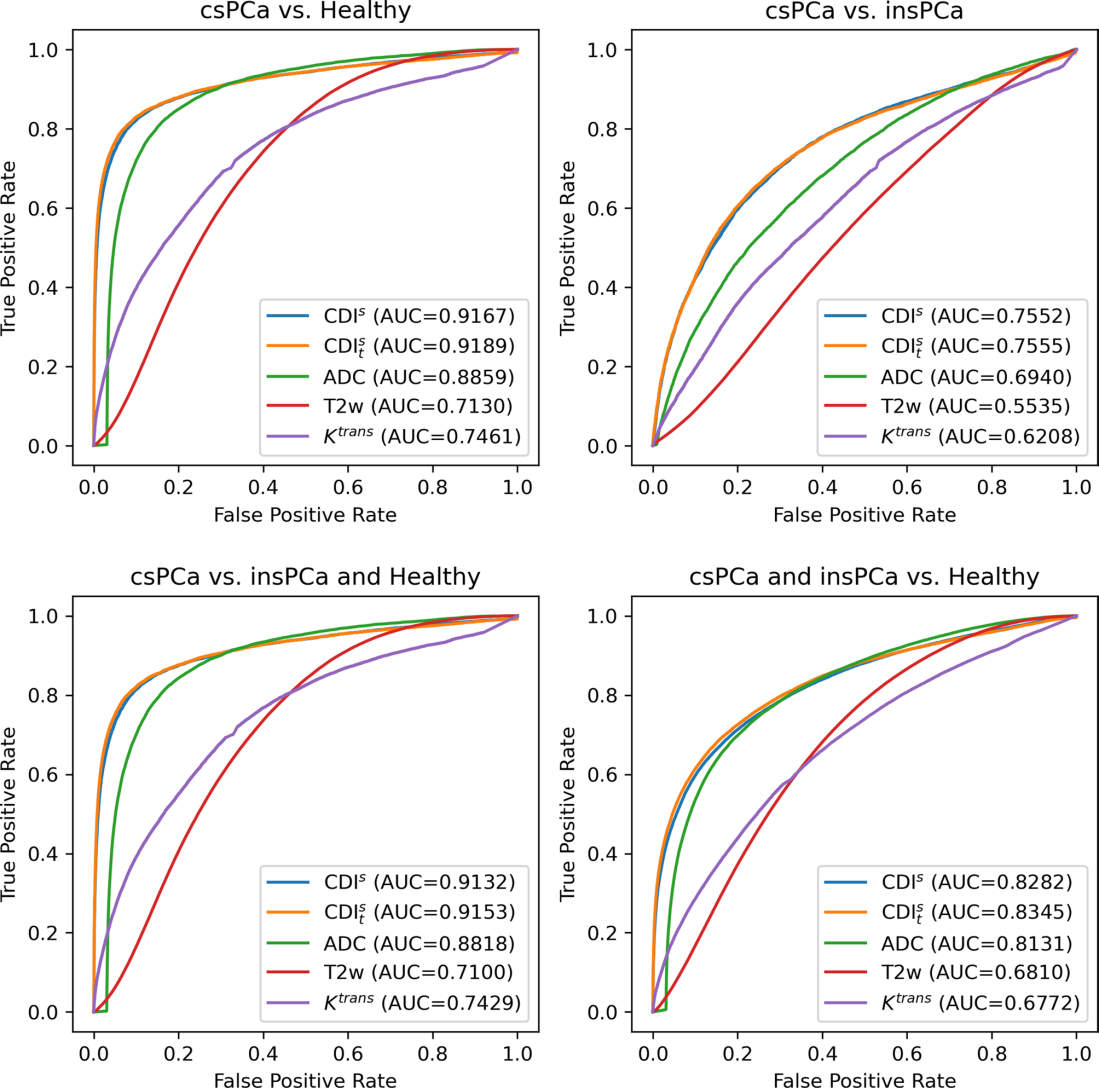
ROC curves for studying the performance of CDI$^s$ SI, CDI$^s_t$ SI, T2w SI, DWI-derived ADC values, and DCE-derived $K^{trans}$ values for delineating csPCa tissue and insPCa tissue from healthy tissue.

Figure 2 shows the ROC curves for studying the performance of CDI$^s$ SI, CDI$^s_t$ (tuned CDI$^s$) SI, T2w SI, DWI-derived ADC values, and DCE-derived $K^{trans}$ values for delineating csPCa tissue and insPCa tissue from healthy tissue. Differences in area under the curve (AUC) between modalities were assessed for statistical significance using the formulation proposed by Hanley and McNeil^22^, with the results of these tests given in Supplementary Table 1.

A number of observations can be made from this ROC analysis. First, it can be observed that CDI$^s$ SI and CDI$^s_t$ SI achieve noticeably higher AUC for delineating between csPCa tissue and healthy tissue when compared to the current standard MRI techniques, with DWI-derived ADC values achieving the next highest AUC (lower by $\sim$ 0.0308 when compared to CDI$^s$ SI, $p<0.0001$). T2w and DCE-derived $K^{trans}$ values achieve significantly lower AUC compared to the other techniques (lower by as much as $\sim$ 0.2037 when compared to CDI$^s$ SI, $p<0.0001$). When comparing CDI$^s$ SI and CDI$^s_t$ SI, it can be seen that CDI$^s_t$ SI achieves $\sim$ 0.0022 higher AUC ($p=0.0207$) when compared to CDI$^s$ SI, thus illustrating the efficacy of CDI$^s$ coefficient optimization where applicable.

Second, it can be observed that CDI$^s$ SI and CDI$^s_t$ SI achieve significantly greater delineation between csPCa and insPCa tissue when compared to current standard MRI techniques, with DWI-derived ADC values, T2w, and DCE-derived $K^{trans}$ values achieving $\sim$ 0.0612 ($p<0.0001$), $\sim$ 0.2017 ($p<0.0001$), and $\sim$ 0.1344 ($p<0.0001$) lower AUC when compared to CDI$^s$ SI, respectively. Here, the difference in delineation performance between csPCa and insPCa tissue for CDI$^s$ SI and CDI$^s_t$ SI is not significant ($p=0.8449$).

Third, it can be observed that CDI$^s$ SI and CDI$^s_t$ SI achieve noticeably higher AUC for delineating between csPCa tissue and other tissue when compared to the current standard MRI techniques, with DWI-derived ADC values achieving the next highest AUC (lower by $\sim$ 0.0314 when compared to CDI$^s$ SI, $p<0.0001$). T2w and DCE-derived $K^{trans}$ values achieve significantly lower AUC compared to the other techniques (lower by as much as $\sim$ 0.2032 when compared to CDI$^s$ SI, $p<0.0001$). When comparing CDI$^s$ SI and CDI$^s_t$ SI, it can be seen that CDI$^s_t$ SI achieves $\sim$ 0.0021 higher AUC ($p=0.0251$) when compared to CDI$^s$ SI, thus again illustrating the efficacy of CDI$^s$ coefficient optimization where applicable.

Fourth, it can be observed that CDI$^s$ SI and CDI$^s_t$ SI achieve noticeably higher AUC for delineating between PCa tissue (both csPCa and insPCa) and healthy tissue when compared to the current standard MRI techniques, with DWI-derived ADC values achieving the next highest AUC (lower by $\sim$ 0.0151 when compared to CDI$^s$ SI, $p<0.0001$). T2w and DCE-derived $K^{trans}$ values achieve significantly lower AUC compared to the other techniques (lower by as much as $\sim$ 0.1510 when compared to CDI$^s$ SI, $p<0.0001$). When comparing CDI$^s$ SI and CDI$^s_t$ SI, it can be seen that CDI$^s_t$ SI achieves $\sim$ 0.0063 higher AUC ($p<0.0001$) when compared to CDI$^s$ SI, thus again illustrating the efficacy of CDI$^s$ coefficient optimization where applicable.

### Clinical interpretation

**Figure 3:**
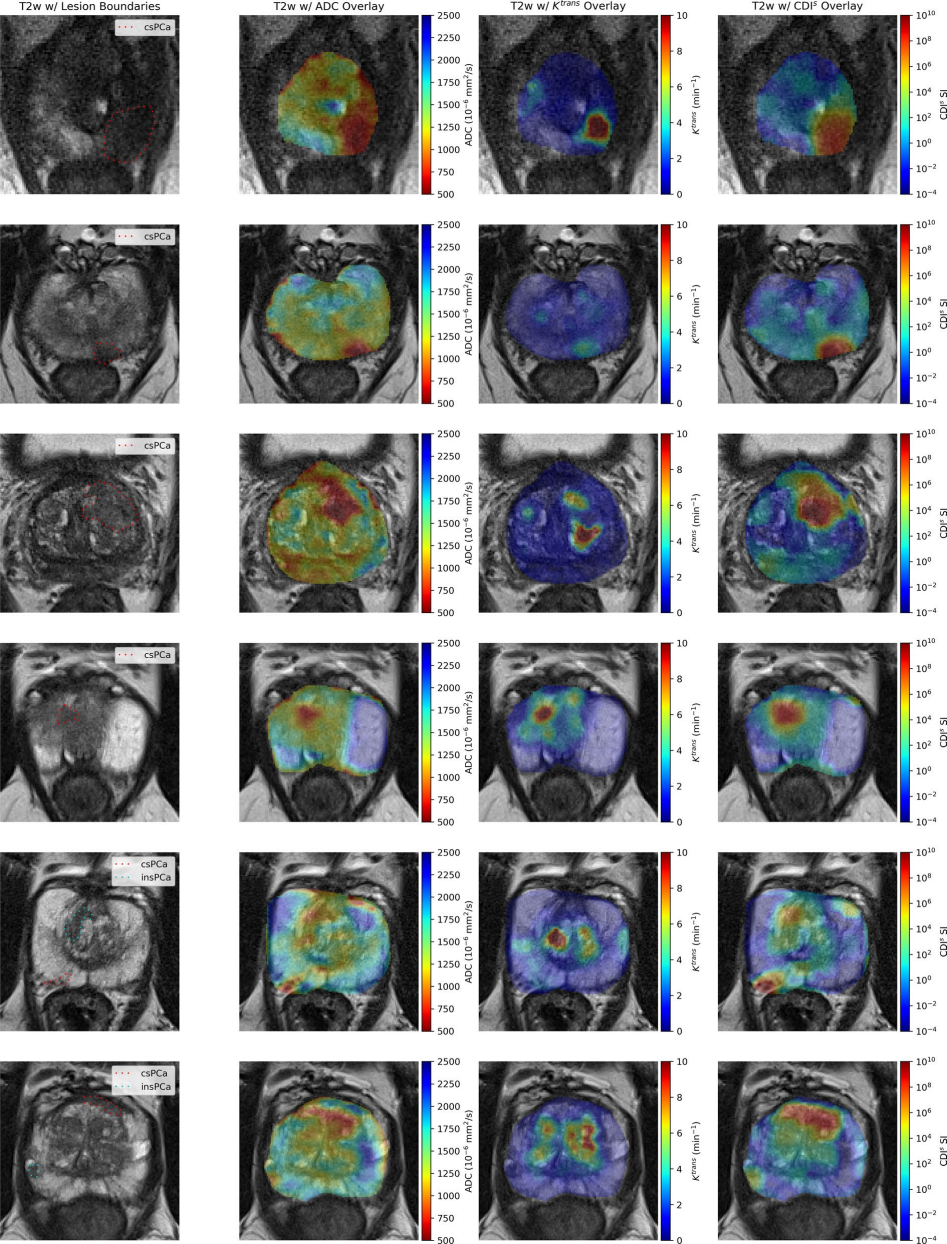
T2w images with overlays of lesion boundaries, DWI-derived ADC, DCE-derived $K^{trans}$, and CDI$^s$ for six patient cases. (**a**, **b**) Two patients with csPCa in the PZ. (**c**, **d**) Two patients with csPCa in the TZ. (**e**) A patient with csPCa in the PZ and insPCa in the TZ. (**f**) A patient with csPCa in the AS and insPCa in the PZ.

Figure 3a, b shows the T2w and overlays of DWI-derived ADC, DCE-derived $K^{trans}$, and CDI$^s$ for two patient cases with csPCa in the PZ. In Fig. 3a, it can be observed that T2w shows no contrast between csPCa tissue and healthy tissue, while $K^{trans}$ exhibits strong contrast for a smaller portion within the csPCa tumor. ADC shows good contrast between the csPCa tumor and some of the surrounding healthy tissue, but exhibits ADC values similar to the tumor in different small regions within the TZ, including an adjacent region above the tumor which is indistinguishable from the tumor itself. CDI$^s$ shows strong contrast for the entire csPCa tumor from the rest of the healthy tissue.

In Fig. 3b, it can be observed that T2w shows poor contrast between csPCa tissue and healthy tissue, while $K^{trans}$ exhibits poor contrast between the csPCa tumor and healthy tissue. ADC shows good contrast between the csPCa tumor and surrounding healthy tissue, but exhibits ADC values similar to the tumor in another small region within the PZ that was not identified as PCa tissue. CDI$^s$ shows strong contrast for the entire csPCa tumor from the rest of the healthy tissue.

Figure 3c, d shows the T2w and overlays of DWI-derived ADC, DCE-derived $K^{trans}$, and CDI$^s$ for two patient cases with csPCa in the TZ. In Fig. 3c, it can be observed that T2w shows no contrast between csPCa tissue and healthy tissue. $K^{trans}$ exhibits mild contrast in a small region within the csPCa tumor, but strong contrast in a healthy tissue region that is not associated with csPCa. ADC shows strong contrast for the csPCa tumor from surrounding tissue, but exhibits ADC values similar to other regions within the PZ that were not identified as PCa tissue via histopathology validation. CDI$^s$ shows strong contrast for the entire csPCa tumor from the rest of the healthy tissue. In Fig. 3d, it can be observed that T2w shows no contrast between csPCa tissue and healthy tissue, while $K^{trans}$, ADC, CDI$^s$ all exhibit strong contrast between csPCa tissue and healthy tissue.

Figure 3e shows the T2w and overlays of DWI-derived ADC, DCE-derived $K^{trans}$, and CDI$^s$ for a patient with csPCa in the PZ and insPCa in the TZ. It can be observed that T2w shows no contrast between the csPCa tumor and healthy tissue, and poor contrast between the insPCa tumor and healthy tissue. $K^{trans}$ exhibits no contrast between the csPCa tumor and healthy tissue, and strong contrast for a small portion of the insPCa tumor. ADC shows strong contrast between the csPCa tumor and surrounding healthy tissue and good contrast between the insPCa tumor and surrounding healthy tissue. However, ADC exhibits similar values for both the csPCa and insPCa tumors, as well as ADC values similar to the tumors in small regions within the TZ that were not identified as PCa tissue. CDI$^s$ show the strongest contrast between the csPCa tumor and healthy tissue amongst the techniques, and shows good contrast between the insPCa tumor and healthy tissue. Furthermore,CDI$^s$ provides greater contrast between the csPCa tumor and insPCa tumor than ADC.

Figure 3f shows the T2w and overlays of DWI-derived ADC, DCE-derived $K^{trans}$, and CDI$^s$ for a patient with csPCa in the anterior stroma (AS) and insPCa in the PZ. It can be observed that T2w shows poor contrast between the csPCa tumor and healthy tissue, with T2w SI of the csPCa tumor being similar to healthy tissue in the TZ. T2w also shows good contrast between the insPCa tumor and healthy tissue, although T2w SI of the insPCa tumor is similar to healthy tissue in the TZ. Furthermore, the T2w SI of the csPCa tumor is very similar to that of the insPCa tumor. $K^{trans}$ exhibits no contrast between the csPCa tumor and healthy tissue and no contrast between the insPCa tumor and healthy tissue. Furthermore, $K^{trans}$ exhibits contrast in healthy tissue in the TZ that is not identified as PCa. ADC shows poor contrast between the csPCa tumor and surrounding healthy tissue and good contrast between the insPCa tumor and surrounding healthy tissue. CDI$^s$ show the strongest contrast between the csPCa tumor and healthy tissue amongst the techniques, and shows good contrast between the insPCa tumor and healthy tissue. Furthermore, CDI$^s$ provides greater contrast between the csPCa tumor and insPCa tumor than ADC.

## Discussion

In this study, we hypothesised that there is a strong relationship between SI of CDI$^s$ and the presence of PCa, and the experimental results support this hypothesis. Results across a cohort of 200 patient cases with histopathology validation showed that hyperintensity in CDI$^s$ provides a strong indicator of the presence of csPCa. Furthermore, CDI$^s$ achieves strong delineation of clinically significant cancerous tissue and healthy tissue (AUC exceeding 0.918 and 0.916 for CDI$^s_t$ and CDI$^s$, respectively), which is noticeably higher than current standard techniques for prostate screening such as T2w, DWI, and DCE. In general, CDI$^s$ also shows fewer false positive regions compared to the other techniques. These results suggest that the use of CDI$^s$ may have a clinical impact as a diagnostic aid for improving PCa screening.

To improve diagnostic accuracy when using MRI for PCa screening and diagnosis, DWI is often used alongside T2w, with DWI being the primary determining modality for the PZ in PI-RADS^12^. In DWI, pairs of opposing magnetic field gradient pulses are applied in the imaging sequence to obtain sensitivity to the Brownian motion of water molecules in tissues^23,24^. Therefore, given the presumed higher cellular density of cancerous tissue compared to non-cancerous tissue, potentially cancerous regions would exhibit markedly reduced ADC^12,24^ due to restricted diffusion. Despite its considerable promise^25–29^, the use of DWI for PCa screening and diagnosis remains a challenge due to considerable ADC variability depending on the strength, duration, and timing of the applied diffusion gradient pulses used in the DWI pulse sequences, thus necessitating tuning of these parameters. This is further complicated by significant overlap in ADC between stromal benign prostatic hyperplasia (BPH), anterior fibromuscular stroma (AFMS), central zone (CZ) tissue, and PCa^30,31^.

Another well-established modality leveraged to improve diagnostic accuracy when using MRI for PCa screening and diagnosis is DCE imaging^32^. Here, a contrast agent (low molecular-weight gadolinium chelate) is injected intravenously, and T1-weighted MRI (T1w) is acquired before, during, and after the injection. Given the increased permeability of the tumor vessels, potentially cancerous regions would exhibit a high volume transfer constant between blood plasma and the extravascular extra-cellular space (denoted by $K^{trans}$). The use of DCE for PCa screening and diagnosis remains a challenge due to sensitivity to patient motion, lack of specificity^32^, and additional acquisition complexities such as cost and process overhead due to the use of an agent. As such, DWI, T2w, and DCE are frequently used together in the form of mpMRI to overcome the shortcomings of each modality; however, the need to interpret multiple modalities also increases the difficulty in interpretation, leading to increased inter- and intra-observer variability.

To address the aforementioned shortcomings of DWI and ADC maps for PCa screening and diagnosis, a new diffusion MRI modality was recently introduced in the form of CDI^19^. In CDI, a series of pulse sequences with different gradient pulse strengths and timings are used to probe water molecules with different degree of Brownian motion in the tissues within a local volume. Signal mixing is then performed on the signal acquisitions captured using these pulse sequences to determine the joint correlation of the acquisitions within a local volume. As such, CDI leverages the distribution of water molecules with different degrees of Brownian motion in the tissues within the local volume to delineate between cancerous tissue (indicated by signal hyperintensity due to a wider spread in the distribution of water molecules with varying degrees of Brownian motion within a local volume) and non-cancerous tissue (indicated by lower relative intensity due to a tighter distribution of water molecules with a similar degree of Brownian motion within a local volume). While preliminary studies have shown that CDI holds considerable promise of achieving greater signal delineation between cancerous and non-cancerous tissue when used as a standalone diagnostic imaging method^19^ and when used in combination with T2w and DWI^20^, these studies are rather limited in scope as the patient study sizes and patient diversity was relatively small. Furthermore, CDI as it was originally investigated has several limitations associated with acquisition time and SNR-associated restrictions, and variability in SI amongst inter-patient and intra-patient acquisitions. As such, a comprehensive study with a significantly larger patient size as well as extensions to CDI to address the aforementioned limitations is highly desired to achieve a thorough investigation and evaluation on the relationship between signal hyperintensity in CDI and presence of PCa, which was the basis of this study.

In conclusion, our results in this study support the hypothesis that the use of CDI$^s$ can be an effective tool for PCa screening and diagnosis, although additional studies are needed before adoption for routine clinical use. Furthermore, given the promising results, we aim to investigate the relationship of CDI$^s$ SI and the presence of other forms of cancer such as breast cancer, gastric cancer, and glioblastoma.

## Methods

### Imaging protocol

To study the relationship between CDI$^s$ SI and PCa, a cohort of 200 patient cases with histopathology validation acquired at Radboud University Medical Centre (Radboudumc) in the Prostate MRI Reference Center in Nijmegen, The Netherlands^33^ were used in this study. Notably, findings with a PI-RADS score of 1 or 2 were not biopsied and were considered clinically insignificant. Table 1 summarizes the demographic, MR scanner, and clinical significance variables of the patient cohort used in this study. The patients in this cohort ranged in age from 37-78 years, with a median age of 64 years. All acquisitions were performed using a Siemens MAGNETOM Trio 3.0T machine or a Siemens MAGNETOM Skyra 3.0T machine, and were reviewed by or performed under the supervision of an expert radiologist with over 20 years of experience interpreting prostate MRI^33^.

A single-shot echo-planar sequence was used for axial DWI acquisitions, with TR ranging from 2500 to 3300 ms with a median of 2700 ms and TE ranging from 63 to 81 ms with a median of 63 ms. The in-plane resolution of the acquisitions was 2 mm with slice thickness ranging from 3 to 4.5 mm with a median of 3 mm. The display field of view (DFOV) ranged from $16.8\times 25.6$ to $24.0\times 25.6$ cm$^2$ with a median of $16.8\times 25.6$ cm$^2$, and images were acquired at *b*-values of $50~\text {s/mm}^2$, $400~\text {s/mm}^2$, and $800~\text {s/mm}^2$. To compare the performance of CDI$^s$ for PCa delineation with current standard MRI techniques, ADC maps were also obtained from DWI acquisitions.

Axial T2w acquisitions were also obtained as a reference of comparison, and were performed using a turbo spin-echo sequence with TR ranging from 3880 to 7434.8 ms with a median of 5660 ms and TE ranging from 101 to 112 ms with a median of 104 ms. The in-plane resolution of the acquisitions ranged from 0.3 to 0.6 mm with a median of 0.5 mm and slice thickness ranged from 3 to 4.5 mm with a median of 3 mm. The DFOV ranged from $18\times 18$ cm$^2$ to $19.2\times 19.2$ cm$^2$ with a median of $19.2\times 19.2$ cm$^2$.

Finally, axial DCE imaging was conducted with a turbo flash gradient-echo sequence, with TR ranging from 3.72 to 36 ms with a median of 36 ms and TE ranging from 1.41 to 1.84 ms with a median of 1.41 ms. The in-plane resolution of the acquisitions ranged from 1.3 to 1.8 mm with a median of 1.5 mm, slice thickness ranged from 3 to 5 mm with a median of 3.5 mm, and the temporal resolution was 3.5 s. The DFOV ranged from $19.2\times 19.2$ cm$^2$ to $25\times 25$ cm$^2$ with a median of $19.2\times 19.2$ cm$^2$. Maps of the pharmacokinetic parameter $K^{trans}$ were obtained from the DCE series.

PCa, whole gland, transition, and PZ annotations for all patient acquisitions in this cohort were used in this study, with the annotation being performed by two radiology residents and two experienced board-certified radiologists (working in pairs) at the University of Naples Federico II, Naples, Italy^34^. Clinical interpretation of CDI$^s$, T2w, ADC, and $K^{trans}$ was conducted in this study by an expert radiologist with over 20 years of experience interpreting prostate MRI (MAH).

**Table 1:** Summary of demographic, MR scanner, and clinical significance variables of the patient cohort used in this study. Age and MR scanner statistics are expressed on a patient level, while clinical significance statistics are expressed on a tumor level.

**Age**	
30–39	3 (1.5%)
40–49	5 (2.5%)
50–59	45 (22.5%)
60–69	112 (56%)
70–79	35 (17.5%)
**MR Scanner (Siemens MAGNETOM)**	
Skyra 3.0T	195 (97.5%)
Trio 3.0T	5 (2.5%)
**Clinical significance (Gleason Score)**	
csPCa (GS $\ge$ 7)	76 (25.4%)
insPCa (GS < 7 or PI-RADS^12^ $\le$ 2)	223 (74.6%)

### Synthetic correlated diffusion imaging

In this study, an extended variant of CDI we term CDI$^s$ is introduced. The first key distinguishing aspect of CDI$^s$ when compared to CDI is the introduction of synthetic signal acquisitions alongside native signal acquisitions to reduce acquisition time, allow existing clinical imaging protocols and pulse sequences that are routine in mpMRI imaging sessions to be used, as well as overcome SNR limitations and distortion limitations faced by CDI, particularly under gradient pulse configurations with longer echo times. The second key distinguishing aspect of CDI$^s$ when compared to CDI is the introduction of signal calibration into the signal mixing procedure of CDI to allow for greater consistency in the resulting SI dynamic range across machines and protocols. This signal calibration thus addresses the issue associated with CDI with respect to large variability in SI amongst inter-patient and intra-patient acquisitions that could highly affect clinical interpretation.

**Figure 4:**
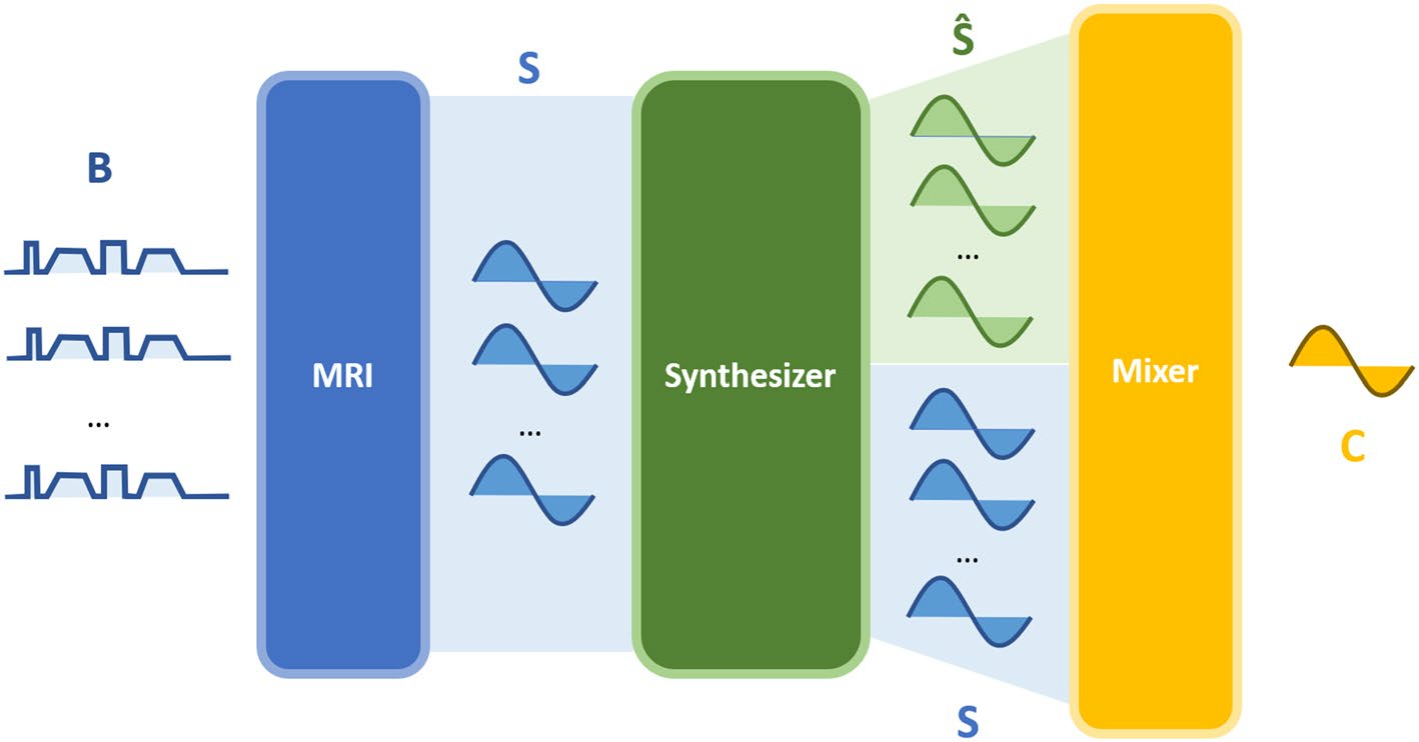
The methodology behind synthetic correlated diffusion imaging, which can be summarized as follows. First, multiple native DWI acquisitions are performed using a set of *b*-values $B=\{b_1, b_2, ..., b_N\}$. Second, the native signal acquisitions *S* are passed into a signal synthesizer to compute synthetic signal acquisitions ${\hat{S}}$. Third, the native and synthetic signal acquisitions are mixed together in a calibrated manner to obtain the local correlation of signal attenuation across the acquired signals, which produces a final signal (C) that characterizes the tissue being imaged with greater consistency in dynamic range across machines and protocols.

The methodology behind CDI$^s$ is summarized in Fig. 4. First, multiple DWI signal acquisitions are conducted using a set of different configurations of gradient pulse strengths and timings. By varying these configurations between signal acquisitions, each acquisition is sensitized to a different degree of Brownian motion, allowing the multiple signal acquisitions to provide a more complete picture of the tissue characteristics within a local volume by quantifying the distribution of water molecules with respect to their degree of Brownian motion within tissue. The different configurations of gradient pulse strengths and timings used in CDI$^s$ can be represented by a set of so-called *b*-values, denoted $B=\{b_1, b_2, ..., b_N\}$. Each *b*-value $b_i$ may be expressed as

1 $$\begin{aligned} b_i=\gamma ^2 G_i^2\delta _i^2 \big ( \Delta _i-\frac{\delta _i}{3}\big ), \end{aligned}$$

where $\gamma$ denotes the proton gyromagnetic ratio, $G_i$ represents the gradient pulse strength, $\delta _i$ represents the gradient pulse duration, and $\Delta _i$ represents the time between gradient pulses^35^.

Second, the multiple acquired signals are then passed into a signal synthesizer to synthesize synthetic signal acquisitions at desired configurations of gradient pulse strengths and timings not captured via native signal acquisitions. Third, the native and synthetic signal acquisitions are mixed together in a calibrated manner to obtain the final quantitative signal characterizing the joint correlation across the acquired signals within a local volume *V*. The idea behind this calibrated signal mixing procedure in CDI$^s$ stems from our hypothesis that the distribution of water molecules with different degrees of Brownian motion within tissue in a local volume with PCa would differ significantly from that with non-cancerous tissue. For example, healthy PZ regions are largely comprised of glandular tissue, resulting in tighter distributions characterized by high relative SI at gradient pulse configurations with lower *b*-values and low relative SI at gradient pulse configurations with higher *b*-values used in CDI$^s$. More importantly, BPH, AFMS, and CZ are all non-cancerous and are largely comprised of non-glandular tissue, resulting in distributions characterized by low relative SI across all gradient pulse strengths and timings used in CDI$^s$. On the other hand, PCa regions are characterized by a more heterogeneous mixture of tissue with various degrees of relatively high cellular densities, resulting in a wider spread in the distribution of water molecules with varying degrees of Brownian motion within a local volume, including slow moving water molecules due to various degrees of true restricted diffusion, and thus high relative SI across all gradient pulse strengths and timings used in CDI$^s$. In the case of DWI, ADC hypointensity is exhibited by BPH, AFMS, CZ, and PCa, resulting in significant ADC overlaps between them (particularly depending on the choice of DWI sequence) and thus increased risk of false positives^30^. By taking advantage of these distribution differences between PCa and non-cancerous tissue in the signal mixing process in the form of joint correlation in a calibrated fashion, CDI$^s$ can achieve improved signal contrast between PCa and non-cancerous tissue and facilitate for more effective PCa screening and diagnosis, as well as greater consistency in SI dynamic range across machines and protocols to reduce inter-patient and intra-patient variability in clinical interpretation.

As mentioned earlier, we extend upon the signal mixing process in CDI^19^ in two key ways to form CDI$^s$. First, we introduce a calibrated signal mixing function $C({\underline{x}})$ for characterizing the contribution-adjusted local signal correlation across the multiple signal acquisitions, which is parameterized by a set of *b*-values $\{b_{\alpha },...,b_{\beta }\}$ and is defined as

2 $$\begin{aligned} C_{\{b_{\alpha },...,b_{\beta }\}}({\underline{x}})=\frac{1}{Z}\int \dots \int {S_{b_{\alpha }}({\underline{x}}')^{\rho _\alpha }\dots S_{b_{\beta }}({\underline{x}}')^{\rho _\beta }}f\left( S_{b_{\alpha }}({\underline{x}}'),\dots ,S_{b_{\beta }}({\underline{x}}')|V({\underline{x}})\right) dS_{b_{\alpha }}({\underline{x}}') \dots dS_{b_{\beta }}({\underline{x}}'), \end{aligned}$$

where ${\underline{x}}$ represents spatial location, *S* represents a signal acquisition, *f* presents the conditional joint probability density function, $V({\underline{x}})$ represents a local volume around ${\underline{x}}$, $\rho _i$ represents coefficients for controlling the contribution of the different gradient pulse strengths and timings, and *Z* represents a calibration factor. The calibration factor allow the signal mixing function to compensate for inherent variations due to the scanner machine and imaging protocol used during acquisition which can lead to differences in CDI SI appearance across patients or even for the same patient at different acquisitions.

For this study, $\{b_{\alpha },...,b_{\beta }\}$ was set at $\{50~\text {s/mm}^2,1000~\text {s/mm}^2,...,7000~\text {s/mm}^2\}$ (at $1000~\text {s/mm}^2$ intervals), *V* was defined as a 6 mm $\times$ 6 mm $\times$ 3 mm volume centered at ${\underline{x}}$, and the definition of $\rho$ will be discussed in a later section. The probability density function *f* was defined as an uncorrelated Gaussian distribution with mean ${\underline{x}}$ and covariance matrix $\Sigma =\mathbf{diag} (4~\text {mm}^2,4~\text {mm}^2,0~\text {mm}^2)$, and the calibration factor *Z* is computed as the median CDI$^s$ SI within the prostate gland. These definitions yield the specific form of Equ. (2) used in this study:

3 $$\begin{aligned} C_{\{b_{\alpha },...,b_{\beta }\}}({\underline{x}})=\frac{1}{Z}\underset{V({\underline{x}})}{\iiint } {S_{b_{\alpha }}({\underline{x}}')^{\rho _\alpha }\dots S_{b_{\beta }}({\underline{x}}')^{\rho _\beta }}f\left( {\underline{x}}';{\underline{x}}, \Sigma \right) d{\underline{x}}' \end{aligned}$$

Second, we introduce the hybrid use of both native and synthetic signal acquisitions in the signal mixing process, thus leading to the notion of CDI$^s$. The use of synthetic signal acquisitions alongside native signal acquisitions enables us to reduce the acquisition time required to capture signals at gradient pulse configurations with higher *b*-values, as well as overcome SNR limitations with acquiring signals at gradient pulse configurations with higher *b*-values due to factors such as longer echo times and eddy current-induced distortions. Furthermore, it enables the leveraging of existing clinical imaging protocols and pulse sequences that are routine in mpMRI imaging sessions. More specifically, a synthetic signal acquisition ${\hat{S}}_b$ at gradient pulse configuration w ith a particular *b*-value can be synthesized at the signal synthesizer as

4 $$\begin{aligned} {\hat{S}}_b({\underline{x}}) = S_{b_{ref}}({\underline{x}}) \exp \left( -(b-b_{ref}){\hat{A}}({\underline{x}})\right) , \end{aligned}$$

where $S_{b_{ref}}$ is a reference signal acquisition at $b=b_{ref}$ and ${\hat{A}}({\underline{x}})$ is the least-squares ADC estimate. ${\hat{A}}({\underline{x}})$ is given by Equ. (5), where ${\overline{b}}$ and $\overline{\log (S)}$ are the average *b*-value and average log-intensity of the acquired native signals, respectively:

5 $$\begin{aligned} {\hat{A}}({\underline{x}}) = -\frac{\sum \limits _{b_i\in B}(b_i-{\overline{b}})\left( \log (S_{b_i})-\overline{\log (S)}\right) }{\sum \limits _{b_i\in B}(b_i-{\overline{b}})^2} \end{aligned}$$

In this study, native signal acquisitions $S_{b_i}$ at $B=\{50~\text {s/mm}^2400~\text {s/mm}^2800~\text {s/mm}^2\}$ were leveraged to synthesize the aforementioned synthetic signal acquisitions ${\hat{S}}$ at $\{1000~\text {s/mm}^2,...,7000~\text {s/mm}^2\}$ (at $1000~\text {s/mm}^2$ intervals). The synthesized signal acquisitions were then used alongside the native signal acquisition at $b=50~\text {s/mm}^2$ in the signal mixing process for CDI$^s$.

### CDI$^s$ coefficient optimization

As described in Equ. (2), the contribution of different gradient pulse strengths and timings to the CDI$^s$ signal produced by the signal mixer can be controlled via coefficients $\rho$. In this study, we will study the efficacy of CDI$^s$ in both a baseline form (i.e., $\rho =1$ for all gradient pulse strengths and timings) as well as in a form that is tuned specifically to optimize delineation. More specifically, we tune the coefficients $\rho$ for the different gradient pulse strengths and timings using a Nelder-Mead simplex optimization strategy, with the objective function being the area under the ROC curve. This coefficient optimization will yield a tuned form of CDI$^s$, which we denote CDI$^s_t$, that accounts for the importance of the different gradient pulse strengths and timings to delineation performance.

### Visualization of CDI$^s$

To map CDI$^s$ SI in a form that is more natural for clinical interpretation, the CDI$^s$ SI is transformed to the logarithmic space for clinical visualization purposes. Given that CDI$^s$ is computed as a product of exponential signal acquisitions, CDI$^s$ SI is more naturally interpreted in a logarithmic space. Furthermore, the transformed CDI$^s$ SI is visualized as a heatmap overlay on T2w images to provide additional anatomical context with respect to the prostate gland. All image visualizations of CDI$^s$ SI in this study are shown with the aforementioned transforms. Finally, DWI-derived ADC and DCE-derived $K^{trans}$ are visualized as heatmap overlays on T2w images for comparison purposes in this study.

### Statistical analysis

Two different analysis methods were utilized in this study to investigate the relationship between CDI$^s$ SI and the presence of PCa, as well as the performance of CDI$^s$ for delineating between csPCa tissue, insPCa tissue, and healthy tissue. In the first analysis method, we study the relationship between CDI$^s$ SI and the presence of PCa by performing histogram analysis to study the distribution of CDI$^s$ SI for healthy tissue, csPCa tissue, and insPCa tissue. In the second analysis method, a receiver operating characteristic (ROC) curve analysis was performed using CDI$^s$ to quantitatively assess the ability to delineate between healthy tissue, csPCa tissue, and insPCa tissue. Consistent with the contemporary concept of significant versus insignificant prostate cancer (csPCa vs. insPCa)^21^, csPCa tissue is defined as tissue with a Gleason score greater than or equal to 7 (Gleason Grade Groups 2–5 according to the International Society of Urological Pathology) while insPCa tissue is defined as tissue with a Gleason score less than 7 (Gleason Grade Group 1). The ROC curves were estimated empirically, and for illustrative purposes ROC curves obtained from the pooled data of all patient cases were plotted. To provide a quantitative assessment of diagnostic accuracy, the area under the ROC curve was obtained as a single metric of delineation performance. For comparison purposes, histogram analysis and ROC curve analysis were also performed using T2w, DWI-derived ADC map values, and DCE-derived $K^{trans}$ map values.

To assess the statistical significance of differences in AUC values between different modalities, we adopt the critical ratio formulation of Hanley and McNeil^22^. Specifically, for each pair of modalities, the critical ratio *z* is defined as:

6 $$\begin{aligned} z = \frac{A_1-A_2}{\sqrt{SE_1^2 + SE_2^2 - 2rSE_1 SE_2}}, \end{aligned}$$

where $A_1$ and $SE_1$ denote to the AUC and estimated standard error (SE) of modality 1, $A_2$ and $SE_2$ denote the AUC and estimated SE of modality 2, and *r* denotes the estimated correlation between $A_1$ and $A_2$. The estimated SE for each modality is defined as:

7 $$\begin{aligned} SE_i = \sqrt{\frac{A_i(1-A_i) + (n_P - 1)\left( \frac{A_i}{2 - A_i} - A_i^2\right) + (n_N - 1)\left( \frac{2A_i^2}{1 + A_i} - A_i^2\right) }{n_Pn_N}}, \end{aligned}$$

where $n_P$ and $n_N$ denote the number of true positive and true negative examples, respectively.

To estimate the correlation coefficient *r*, the Pearson product-moment correlation is first used to estimate correlation between the two modalities for positive examples ($r_P$) and negative examples ($r_N$). The average of $r_P$ and $r_N$ and the average of $A_1$ and $A_2$ are then used to linearly interpolate the standard table presented by Hanley and McNeil^22^ to estimate *r*. Notably, differences in image resolution prevent one-to-one comparison of voxels when estimating the Pearson product-moment correlation, and as such the median values for csPCa, insPCa, and healthy tissue are used (computed per-patient).

### Software

Data processing, statistical analysis, and visualization was performed using the Python programming language (version 3.7.5) with the following libraries: OpenCV (version 4.2.0.34)^36^, pydicom (version 2.1.2)^37^, NiBabel (version 3.0.2)^38^, SciPy (version 1.4.1)^39^, scikit-learn (version 0.22.2.post1)^40^, NumPy (version 1.18.2)^41^, SimpleITK (version 2.0.2)^42^, and Matplotlib (version 3.2.1)^43^.

### Ethics

This study has received ethics clearance from the University of Waterloo (30632), and was carried out in accordance with relevant guidelines and regulations. Informed consent was obtained from all subjects and/or their legal guardian(s).

## Supplementary Information

Supplementary Information.

## Data Availability

The MRI dataset analysed during the current study is available in the PROSTATEx repository, https://wiki.cancerimagingarchive.net/display/Public/SPIE-AAPM-NCI+PROSTATEx+Challenges. The corresponding prostate segmentation masks and lesion segmentation masks are available in the PROSTATEx_masks repository, https://github.com/rcuocolo/PROSTATEx_masks.

## Code availability

The code used to compute CDI$^s$ from DWI is available from the corresponding authors on reasonable request.
