# Supplementary material for: Synthetic correlated diffusion imaging hyperintensity delineates clinically significant prostate cancer
Source: Sci Rep. 2022 Mar 1;12:3376. doi: 10.1038/s41598-022-06872-7 (PMC8888633; doi:10.1038/s41598-022-06872-7)
Supplement: Supplementary file 1 — Supplementary Information. [file 41598_2022_6872_MOESM1_ESM.pdf]

Supplementary Table 1. Statistical analysis of differences in area under the ROC curve between all modalities. ROC curve titles correspond to those in Figure 2.

ROC Curve	Modalities	A_1	A_2	r_P	r_N	z	p (two-tailed)
csPCa vs. Healthy	CDI^s, CDI^s_t	0.9167	0.9189	0.9982	0.9802	2.3137	0.0207
	CDI^s, ADC	0.9167	0.8859	0.1886	0.2850	13.0366	0.0000
	CDI^s, T2w	0.9167	0.7130	0.1406	0.0854	113.9934	< 0.0001
	CDI^s, K^{trans}	0.9167	0.7461	0.0057	0.0339	66.0474	< 0.0001
	CDI^s_t, ADC	0.9189	0.8859	0.1994	0.2857	14.0423	< 0.0001
	CDI^s_t, T2w	0.9189	0.7130	0.1464	0.0861	116.4832	< 0.0001
	CDI^s_t, K^{trans}	0.9189	0.7461	0.0041	0.0466	67.2891	< 0.0001
	ADC, T2w	0.8859	0.7130	0.4009	0.6696	100.0295	< 0.0001
	ADC, K^{trans}	0.8859	0.7461	0.1428	0.0295	52.2421	< 0.0001
	T2w, K^{trans}	0.7130	0.7461	0.1922	0.0527	16.4462	< 0.0001
csPCa vs. insPCa	CDI^s, CDI^s_t	0.7552	0.7555	0.9982	0.9823	0.1956	0.8449
	CDI^s, ADC	0.7552	0.6940	0.1886	0.1887	16.3557	< 0.0001
	CDI^s, T2w	0.7552	0.5535	0.1406	0.0037	70.1154	< 0.0001
	CDI^s, K^{trans}	0.7552	0.6208	0.0057	0.1245	37.3343	< 0.0001
	CDI^s_t, ADC	0.7555	0.6940	0.1994	0.1940	16.5100	< 0.0001
	CDI^s_t, T2w	0.7555	0.5535	0.1464	0.0050	70.3044	< 0.0001
	CDI^s_t, K^{trans}	0.7555	0.6208	0.0041	0.1134	37.3052	< 0.0001
	ADC, T2w	0.6940	0.5535	0.4009	0.6527	52.4005	< 0.0001
	ADC, K^{trans}	0.6940	0.6208	0.1428	0.1707	20.4215	< 0.0001
	T2w, K^{trans}	0.5535	0.6208	0.1922	0.0028	27.0110	< 0.0001
csPCa vs. insPCa and Healthy	CDI^s, CDI^s_t	0.9132	0.9153	0.9982	0.9825	2.2405	0.0251
	CDI^s, ADC	0.9132	0.8818	0.1886	0.1881	12.8230	< 0.0001
	CDI^s, T2w	0.9132	0.7100	0.1406	0.0238	110.9176	< 0.0001
	CDI^s, K^{trans}	0.9132	0.7429	0.0057	0.1496	66.6417	< 0.0001
	CDI^s_t, ADC	0.9153	0.8818	0.1994	0.1966	13.8127	< 0.0001
	CDI^s_t, T2w	0.9153	0.7100	0.1464	0.0249	113.1965	< 0.0001
	CDI^s_t, K^{trans}	0.9153	0.7429	0.0041	0.1420	67.6887	< 0.0001
	ADC, T2w	0.8818	0.7100	0.4009	0.6335	97.1469	< 0.0001
	ADC, K^{trans}	0.8818	0.7429	0.1428	0.3370	55.6735	< 0.0001
	T2w, K^{trans}	0.7100	0.7429	0.1922	0.0943	16.4136	< 0.0001
csPCa and insPCa vs. Healthy	CDI^s, CDI^s_t	0.8282	0.8345	0.9959	0.9802	9.3085	< 0.0001
	CDI^s, ADC	0.8282	0.8131	0.1550	0.2850	8.8399	< 0.0001
	CDI^s, T2w	0.8282	0.6810	0.0673	0.0854	108.2148	< 0.0001
	CDI^s, K^{trans}	0.8282	0.6772	0.0442	0.0339	85.7535	< 0.0001
	CDI^s_t, ADC	0.8345	0.8131	0.1600	0.2857	12.5640	< 0.0001
	CDI^s_t, T2w	0.8345	0.6810	0.0728	0.0861	114.2608	< 0.0001
	CDI^s_t, K^{trans}	0.8345	0.6772	0.0434	0.0466	90.1805	< 0.0001
	ADC, T2w	0.8131	0.6810	0.6176	0.6696	114.0235	< 0.0001
	ADC, K^{trans}	0.8131	0.6772	0.2279	0.0295	79.4537	< 0.0001
	T2w, K^{trans}	0.6810	0.6772	0.0054	0.0527	3.0826	0.0021
